# Effectiveness of nanoscale zero-valent iron for the immobilization of Cu and/or Ni in water and soil samples

**DOI:** 10.1038/s41598-020-73144-7

**Published:** 2020-09-28

**Authors:** M. Gil-Díaz, M. A. Álvarez, J. Alonso, M. C. Lobo

**Affiliations:** IMIDRA, Instituto Madrileño de Investigación y Desarrollo Rural, Agrario y Alimentario, Finca “El Encín”, Alcalá de Henares, 28805 Madrid, Spain

**Keywords:** Environmental sciences, Environmental impact

## Abstract

In the last few years, the effectiveness of nanoscale zero-valent iron (nZVI) as a treatment for polluted waters and soils has been widely studied. However, little data are available on its efficacy for metal immobilization at low and moderate doses. In this study, the effectiveness of two doses of commercial nZVI (1 and 5%) to immobilize Cu and/or Ni in water and acidic soil samples was evaluated. The influence of the nanoremediation technology on iron availability, physico-chemical soil properties and soil phytotoxicity was also assessed. The results show that the effectiveness of nZVI to immobilize Cu and Ni in water and soil samples was determined by the dose of the nanomaterial and the presence of both metals. Nickel immobilization was significantly decreased by the presence of Cu but the opposite effect was not observed. nZVI showed better immobilization capacity in water than in soil samples. In water, the dose of 5% completely removed both metals, whereas at a lower dose (1%) the percentage of immobilized metal decreased, especially for Ni in Cu + Ni samples. In soil samples, 5% nZVI was more effective in immobilizing Ni than Cu, with a 54% and 21% reduction of leachability, respectively, in single contaminated samples. In Cu + Ni soil samples, nZVI treatment led to a significant decrease in Ni immobilization, similar to that observed in water samples. The application of nZVI induced a dose-dependent increase in available Fe—a relevant effect in the context of soil rehabilitation. Germination assays of *Medicago sativa* and *Vicia sativa* seeds revealed that treatment with nZVI did not induce phytotoxicity under the experimental conditions tested, and that the phytotoxicity induced by Ni decreased significantly after the treatment. Thus, the use of nZVI emerges as an interesting option for Cu and/or Ni immobilization in water samples. The effectiveness of nZVI to remove Cu from acidic soil samples was moderate, while for Ni it was strongly dependent on the presence of Cu. These observations therefore indicate that the results in water samples cannot be extrapolated to soil samples.

## Introduction

Some of the most common soil pollutants are metals and metalloids, persistent organic pollutants and emerging pollutants. Metals and metalloids are persistent pollutants because they are non-biodegradable as organic pollutants. Some of these elements are essential for plants, animals and humans, but high concentration can cause toxicity^1^. In this context, plants can absorb the metals accumulated in soil and transfer them to the food chain, thereby posing a serious threat to animal and human health. Chronic exposure of humans to heavy metals and metalloids, even at relatively low concentrations, can lead to accumulation of these metals in different parts of the human body, thus impairing health. Soil contamination is usually determined on the basis of the total concentration of metals and metalloids present in the matrix; however, this parameter gives limited information about the potential harmful effect of these pollutants on soil biota. In this regard, metal bioavailability, which refers to the accessible fraction that can be assimilated by an organism, is considered a better indicator of the risk to biota^2^. The bioavailable fraction of metals and metalloids can be estimated by single extraction protocols and sequential extraction methods^3^.

Copper (Cu) and nickel (Ni) are included in the USEPA priority pollutant list. In particular, Cu is a heavy metal with wide industrial applications. It is also present in fertilizers, fungicides and domestic products and is used as an additive in food and drugs. Consequently, several sources have contributed to increase the concentration of Cu in soils. With respect to Ni in soil, the main sources of this heavy metal include: combustion of coal and oil, metal processing plants, and the use of sewage sludge and phosphate fertilizers in agriculture^4^. The mobility of these two metals in soils depends on their chemical characteristics and on soil properties (pH, cation exchange capacity, texture, content and nature of organic matter, redox conditions). In this regard, Cu has a high tendency to be absorbed by organic matter, since humic acids show large capacity to bind to this metal^4^. Carbonates, clay minerals and oxyhydroxides of iron (Fe) and manganese (Mn) also adsorb Cu^4^. Thus, Cu would probably show limited availability in alkaline soils with a moderate organic matter content, whereas it would be relatively mobile in acidic soils containing a low proportion of organic matter. Similarly, the mobility of Ni decreases with pH. Thus, in loamy sandy soils, Ni is slightly mobile and is found mainly in the residual fraction, whereas in soils with a high content of organic matter it can easily form soluble chelates^4^.

Among the strategies most widely used for the remediation of metal-polluted soils, immobilization techniques are considered to be cost-effective and environmentally friendly alternatives to other methods such as vitrification, soil washing, and electrokinetic extraction. Immobilization techniques involve the addition of an agent to the polluted soil in order to reduce the mobility and availability of the metal/s in the matrix, thereby reducing the risk of pollutant uptake by soil biota. In the last few years, the use of nanoscale zero valent iron (nZVI) to reduce the availability of metals and metalloids in water and soil samples has been extensively studied. Experiments at lab-scale using nZVI have obtained satisfactory results in reducing the availability of Ag, Al, As, Be, Cd, Cr, Cu, Hg, Ni, Pb, U, V and Zn in individually polluted water samples^5–13^. In soil samples, the effectiveness of this strategy is greatly determined by soil properties, element characteristics, and type of nZVI used. However, promising results have been reported for As, Cr, Hg, Pb, and Zn immobilization^14–23^. Commercial nZVI has recently been shown to be effective for Cu and Ni immobilization in artificially polluted soil and water samples^24^. Those authors tested doses of 5, 10, 15 and 30% of commercial nZVI. However, doses of 10–30% would be economically and environmentally unsustainable, since a high Fe input could decrease soil pore size, reducing the soil's permeability. Thus, it is necessary to access the effectiveness of nZVI at lower doses, including 1–5%. Limited data are available in the literature on the effectiveness of nZVI at these doses. Moreover, the simultaneous presence of several metal(loid)s may lead to competition between metal(loid) ions, thereby reducing their sorption or producing synergistic effects^6,11,15^. The main objective of the present paper was to evaluate the effectiveness of nZVI to immobilize Cu and Ni in water and acidic soil samples affected by single or dual contamination. The impact of the technology on the physico-chemical properties of the soil, Fe availability, and soil phytotoxicity were also assessed.

## Materials and methods

### Soil

Bulk soil from the surface layer (0–30 cm depth) was collected from an agricultural site located in Daganzo (Madrid). It was air-dried and sieved (< 2 mm) prior to analysis. The physico-chemical properties of the soil were determined following the official methodology in Spain^25,26^ (Table 1). Briefly, pH and electrical conductivity were measured using a 1:2.5 soil:water ratio (w:vol) and 30 min of equilibration time. Total nitrogen was determined by the Kjeldahl method, organic matter by the Walkley–Black method, and soil texture by means of a Bouyoucos densimeter. Available Ca, Mg, Na and K were measured using an atomic absorption spectrometer (AA240FS, Varian) after extraction with ammonium acetate 0.1 N. The total concentration of metals was quantified using atomic absorption spectrometry (AAS), after acid digestion (0.500 g) with a mixture of HNO_3_ (6 mL, 69%) and HCl (2 mL, 37%) in an Anton Paar microwave (Multiwave Go).

**Table 1:** Physico-chemical properties of the soil used in the experiment.

Property	Unit	Soil
pH		5.34
EC	dS/m	0.55
N	%	0.06
OM		0.8
P	mg/kg	40
Ca		598
Mg		90
Na		30
K		155
Fe	mg/kg	1500
Pb		10
Cd		< LD
Cu		9
Cr		9
Zn		30
Ni		< LD
Sand	%	64.3
Silt		25.5
Clay		13.2

EC, electrical conductivity; OM, organic matter; LD, limit of detection.

### Zero valent iron nanoparticles

An aqueous suspension of nZVI Nanofer 25S purchased from NANO IRON s.r.o. (Rajhrad, Czech Republic) was used. The nanoparticle suspension was stabilized with polyacrylic acid, a biodegradable organic surfactant, to avoid the agglomeration phenomena, keeping iron nanoparticles well accessible to reactions. The Fe(0) content was 14–18%, and 2–6% was iron oxides, the average size was close to 60 nm, the active surface area was 20 m^2^/g and the zeta potential was 32 mV^13,22^. Two doses of nZVI (1% and 5%, w:w or 1.8 and 9 g Fe/kg) were selected based on previous studies^15,20,22^ and taking into account that the doses were environmentally and economically viable.

### Batch experiments in water samples

Aqueous solutions of CuSO_4_·5H_2_O (100 mg/L), NiSO_4_·6H_2_O (100 mg/L) (Panreac, Barcelona, Spain) and the mixture of both salts (100 mg/L each one) were prepared in distilled water. Twenty milliliters of metal solutions was placed in a 50 mL-plastic vial and treated with nZVI at two doses (1% and 5%, w:w). The mixtures were shaken for 24, 48 and 72 h in a Reax 2 roller shaker (Heidolph Instrument GmbH & Co. KG). The tubes were then centrifuged at 9000 rpm for 4 min (HITACHI CR 22 N) and the extracts were filtered. Copper and Ni were quantified by atomic absorption spectrometry and compared with control solutions (without addition of nZVI). All experiments were performed in triplicate.

### Batch experiments in soil samples

Soil samples (1 kg) were spiked with Ni and Cu individually and simultaneously, using calculated amounts of CuSO_4_·5H_2_O and/or NiSO_4_·6H_2_O solution (Panreac, Barcelona, Spain), to concentrations of 1000 mg/kg for Cu and 2000 mg/kg for Ni. These concentrations were selected based on the generic reference levels for human health in urban soils, established by Spanish regulations (Orden 2770/2006 CM). The spiked soils were incubated at 25 °C for 30 days at 60% field capacity. After this period, soils were air-dried and sieved (< 2 mm) to homogenize the samples.

The samples were weighed in plastic vials (200 mL), and different doses of nZVI (0%, 1% and 5%, w/w) were applied on them. Distilled water was added to the samples to achieve water holding capacity and to favor soil and nZVI mixing. Vials were shaken at 90 rpm for 72 h in a Rotaterm shaker (Selecta, Barcelona, Spain). The samples were then air-dried before analysis of metal availability, soil properties, and phytotoxicity was carried out. Experiments were carried out in triplicate.

### Metal availability

Two protocols were used to evaluate the effectiveness of metal immobilization: (i) the TCLP (Toxicity Characteristic Leaching Procedure) to determine the potential leachability of Cu and Ni (USEPA Method 1311), based on an extraction with 0.1 M sodium acetate buffer (pH 4.93 ± 0.05); and (ii) the sequential extraction procedure proposed by Tessier, with some modifications to obtain the residual fraction^27^. Tessier’s protocol involves the sequential addition of reagents of increasing strength, and the fractions obtained are (in decreasing order of mobility): exchangeable (EX), bound to carbonate (CB), Fe–Mn oxides (OX), bound to organic matter (OM), and residual (RS). The extracts were filtered before Cu and Ni quantification by atomic absorption spectrometry. In addition, Fe was determined in TCLP and Tessier extracts to evaluate the impact of nZVI application on soil Fe availability.

### Soil phytotoxicity

The phytotoxicity of the nZVI-treated soils was analyzed using the Zucconi test^28^, with some modifications^29^, for two species, namely *Vicia sativa* (common vetch) and *Medicago sativa* (alfalfa). Seven seeds were placed in a Petri dish (9 mm) moistened with 6 mL of soil extract (5 g of soil mixed with 50 mL of distilled water at 60º shaken for 30 min) or distilled water (positive control). After four days of seed incubation at 26–27 ºC in the dark, the germination percentage and root length of seedlings were determined and the GI (germination index) was calculated as follows: GI (%) = G Ls/Lc, where G is the percentage of germination obtained with respect to the control values, Ls is the mean value of the root length in the soil extracts, and Lc is the mean value of the root length in the control.

### Statistical analysis

Data were analyzed by the general linear model (GLM) using the statistical package IBM SPSS version 19.0.0.1. Experimental factors were doses of nZVI and the type of contamination. A post hoc Tukey test was performed to assess which means differed from each other.

## Results and discussion

### Effectiveness of nZVI in water samples

**Figure 1:**
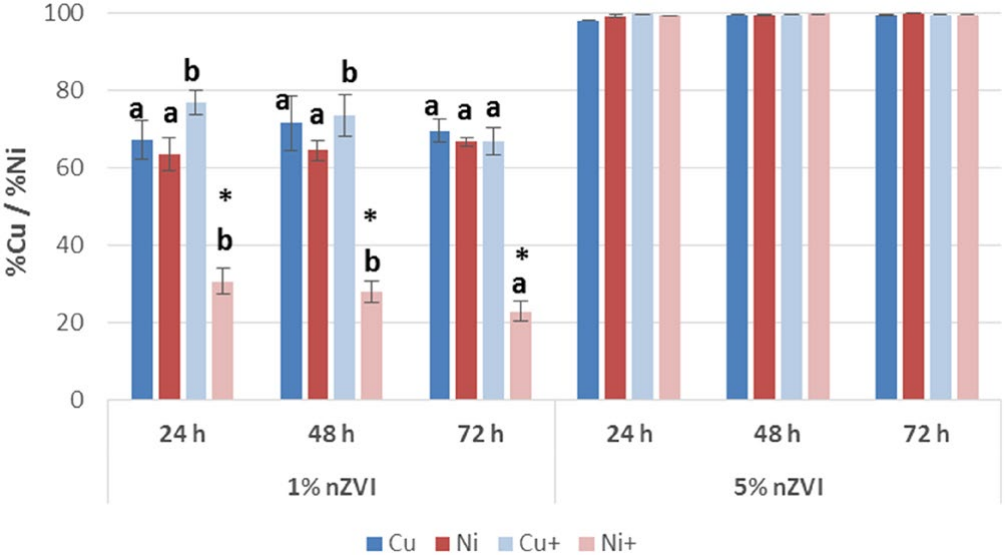
Mean percentage and standard deviation of Cu and/or Ni immobilized at the different conditions. For each type of contamination, bars followed by the same letter do not significantly differ (p < 0.05) in function of the contact time; for each metal, * means significant differences (p < 0.05) between individual and combined contamination.

The results on the effectiveness of nZVI to immobilize Cu and Ni (both individually and in combination) in water samples are shown in Fig. 1. Effectiveness was dose dependent. At the highest dose assayed (5%, 9 g Fe/L), the immobilization of Cu and Ni was almost 100%, even when Cu and Ni were combined. At that dose, no significant differences in immobilization capacity were observed between the times studied. In contrast, at the dose of 1% nZVI (1.8 g Fe/L), immobilization decreased for both metals, especially for Ni in the case of Cu + Ni solutions. This observation can be explained by competition between Cu^2+^ and Ni^2+^. The mean percentages of immobilized Cu and Ni at 1% nZVI were in the range 67–77% and 23–67%, respectively. The presence of Ni did not affect Cu immobilization; however, when Cu was present, Ni removal significantly decreased from 64% (single) to 31% after 24 h of contact time (p < 0.001). In addition, in the case of Cu + Ni contamination, the effectiveness of nZVI to remove the two metals decreased with contact time, reaching 67% and 23% removal of Cu and Ni, respectively after 72 h of interaction. Considering these results, further studies at doses between 1 and 5% of nZVI are necessary. In this regard, Li and Zhang^6^ also observed poor capacity of lab-made nZVI (5 g/L) to immobilize Ni when other metal ions were present, whereas the removal efficiency increased over 80% in individual experiments with Ni^2+^. Vasarevicius et al.^24^ treated aqueous solutions spiked with Cd^2+^, Cu^2+^, Ni^2+^ and Pb^2+^ individually and in combination, and also observed that competition between the metal cations strongly decreased Ni^2+^ removal. Li & Zhang^5^ previously reported that the effectiveness of nZVI was inversely proportional to Ni concentration. The interaction mechanisms between Cu/Ni and nZVI can be explained on the basis of their standard redox potentials, the core–shell structure of the nZVI, its small particle size, large surface area and greater density and adsorptive sites compared with bulk iron^5,6,11,12^. The reduction potential of Cu^2+^ to Cu^0^ (E^0^ = 0.34 V) is more electropositive than Fe (E^0^ = − 0.41 V), so the reduction reaction is thermodynamically favorable (ΔE_h_^0^ = 0.75 V), the predominant mechanism for Cu^2+^ is the reduction to Cu^0^, which precipitates (Eq. 1)^6^. In the case of Ni^2+^, its reduction potential (E^0^ = − 0.24 V) is slightly higher than that of Fe, and both reduction and sorption on the hydrous Fe oxides present in the shell of nZVI can occur. Li & Zhang^5^ concluded that nZVI can act as a sorbent and a reductant for the immobilization of Ni^2+^. The authors applied XPS analysis at different times and concluded that the mechanism includes various stages: firstly, Ni^2+^ is physically adsorbed onto the Fe surface, then a chemisorption process (a specific sorption) with OH^-^ groups performs, and, finally, Ni^2+^ is gradually reduced to Ni^0^. The Eqs. 2–4 represent the surface reactions for Ni^2+^ removal using nZVI (surface complex formation in Eqs. 2 and 3, and surface reduction to Ni^0^ in Eq. 4). The authors found that the treatment of Ni^2+^ solutions (100 mg/L) with 5 g/L nZVI completely removes the metal in 3 h^5^. This mechanism explains that Ni^2+ ^removal was more affected in the presence of another competitive cation such as Cu^2+^, i.e., the effectiveness in water samples polluted with a mixture of both metals and treated with the lowest dose of nZVI (1%), was reduced due to the lower number of available adsorption sites in nanoparticle surface. In contrast, a synergistic effect was detected in the reduction of nitrate to ammonium in water samples due to CuCl_2_ promoted nitrate reduction kinetics^30,31^.

1 $${\text{Fe}} + {\text{Cu}}^{2 + } \to {\text{Fe}}^{2 + } + {\text{Cu}}\quad \Delta E_{{\text{h}}}^{0} = 0.75\,{\text{V}}$$

2 $$\equiv {\text{FeOH}} + {\text{Ni}}^{2 + } \to \equiv {\text{FeO}} - {\text{Ni}}^{ + } + {\text{H}}^{ + }$$

3 $$\equiv {\text{FeONi}}^{ + } + {\text{H}}_{2} {\text{O}} \to \equiv {\text{FeONi}} - {\text{OH}} + {\text{H}}^{ + }$$

4 $$\equiv {\text{FeONi}}^{ + } + {\text{Fe}}^{0} + {\text{H}}^{ + } \to \equiv {\text{FeOH}} - {\text{Ni}} + {\text{Fe}}^{2 + }$$

In summary, the addition of nZVI to water contaminated with Cu and/or Ni appears to be an interesting option for water decontamination. However, when both metals are present, a higher dose of nZVI is necessary to offset competition phenomena. In addition, the nanoremediation process could be designed to recover these valuable metals from contaminated water. In this regard, Li et al.^7^ demonstrated an effective field process for removing Cu^2+^ present in wastewater using nZVI, and they were able to recycle the iron nanoparticles and to recover Cu.

### Effectiveness of nZVI in soil samples

**Figure 2:**
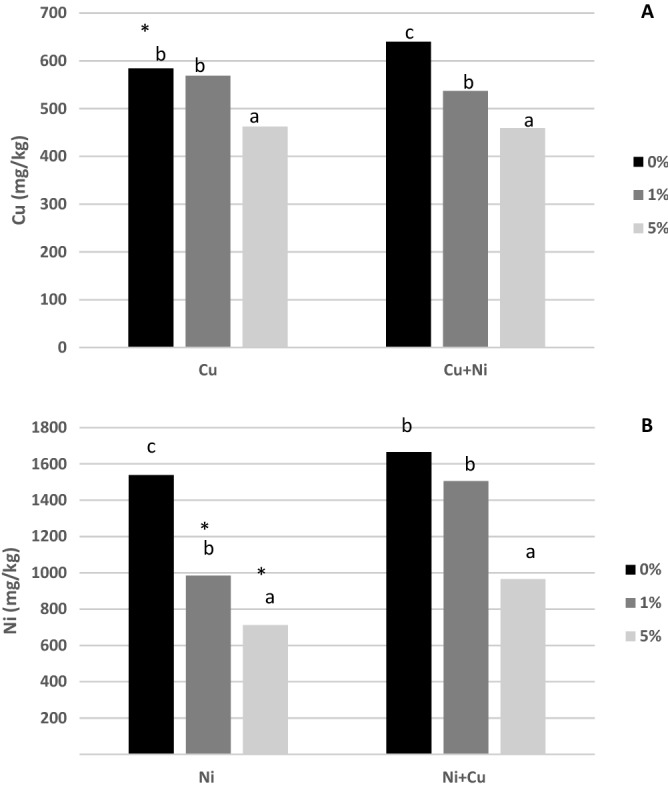
Mean concentration of Cu (mg/kg) (**A**) and Ni (mg/kg) (**B**) in TCLP extract. For each polluted soil, bars followed by the same letter do not significantly differ (p < 0.05); *means significant differences (p < 0.05) between types of contamination for the same dose of nZVI.

The availability of Cu and Ni in soil samples was evaluated by TCLP and Tessier sequential extraction (Fig. 2). In general, the immobilization capacity of nZVI in soil samples was lower than in water samples. In the case of Cu, immobilization was moderate. At the highest nZVI dose, Cu leachability was reduced by 21% (p < 0.001) compared to control soils. In the soils contaminated with Cu + Ni, Cu-TCLP was reduced by 16% and 27% at 1% and 5% nZVI, respectively. Thus, the presence of Ni did not significantly affect the effectiveness of the nanomaterial to remove Cu, as previously observed in water samples. In soil, nZVI showed a greater capacity to immobilize Ni than Cu, with a 36% and 54% reduction of Ni-TCLP at doses of 1% and 5%, respectively. Nevertheless, the presence of Cu significantly reduced the capacity of nZVI to remove Ni. This observation could be attributable to the competitive phenomena due to the presence of the competitive Cu^2+^ cation, a divalent cation which produces an antagonistic effect on Ni^2+^ removal, reducing its immobilization rate as previously explained (see 3.1 section). Significant reduction of Ni-TCLP (42%), was observed only at a dose of 5% nZVI. This result is consistent with those obtained from the water samples, which registered poor immobilization results for Ni in Cu + Ni solutions.

**Figure 3:**
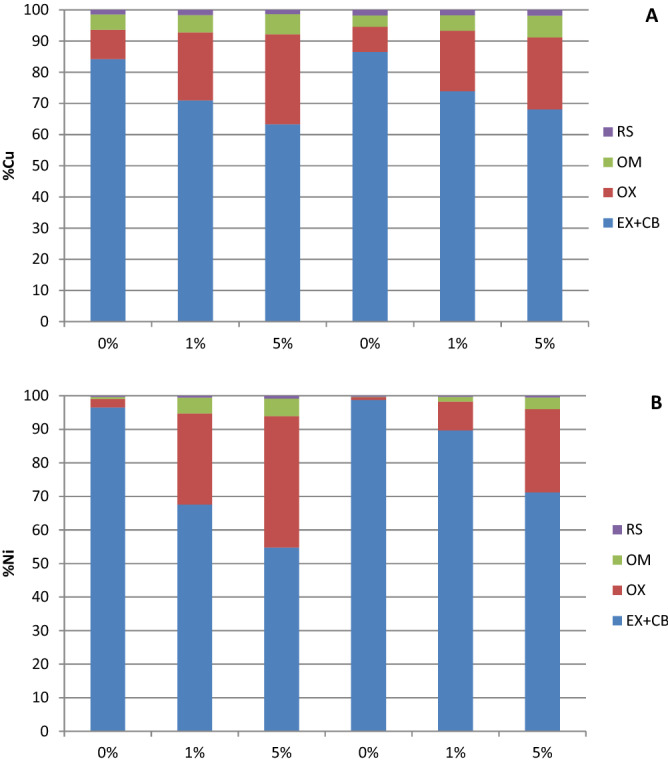
Percentages of Cu (**A**) and Ni (**B**) in each soil fraction, EX + CB (exchangeable + bound to carbonates), OX (bound to oxides), OM (bound to organic matter) and RS (residual), obtained by sequential extraction procedure.

The results obtained for the more available fractions of the Tessier method were consistent with the TCLP results (Fig. 3). The concentration of Cu and Ni associated with the more available fraction (EX + CB) was moderately reduced after application of nZVI. In the case of Cu, the greatest effectiveness was obtained at the highest dose of nZVI (21–28% reduction of Cu in the EX + CB fraction). As previously observed in the TCLP results, immobilization of Ni was better under single contamination conditions, with a reduction of (EX + CB)-Ni of 26% and 44% at doses of 1% and 5% of nZVI, respectively. Nevertheless, in the soil samples polluted with Cu + Ni, the strategy showed a significant decrease in Ni removal capacity, and a Ni reduction of only 32% was achieved with 5% of nZVI. The immobilized Cu and Ni were destined mainly to the oxide fraction, followed by the fraction of organic matter. Copper and Ni in the residual fraction were lower than 1% and 2% respectively, and treatment with nZVI did not significantly increase the metal associated with this fraction.

The moderate effectiveness of nZVI for Cu removal from soils may be explained by the acidic soil pH, with predominant positive charges, thereby increasing the repulsive phenomena with metal cations. In addition, the soil had a low content of organic matter and carbonates, characteristics that enhance Cu mobility. In contrast, Vasarevicius et al.^24^ studied the reduction of the availability of Cd, Cu, Ni and Pb in individually spiked soils or as mixtures, using a dose of 5% of nZVI and over (10, 15 and 30%). The authors noted higher Cu and Ni removal capacity in the case of mixtures of metals and at all the nZVI doses tested. The discrepancy between these results and ours could be explained by the different experimental conditions used, in particular the soil properties and the higher doses of nZVI applied. In addition, the available metal concentration can vary depending on the extraction procedure^3^. Vasarevicius et al.^24^ used CaCl_2_ (0.01 M, 1:10 w:v, 2 h of shaking), whereas we applied TCLP and Tessier fractionation. In general, better immobilization results were achieved in water samples. Therefore, the extrapolation of the results to soil samples would be inappropriate. In this regard, it would be necessary to test this nanoremediation technology in soil samples.

### Impact of nZVI on Fe availability

**Figure 4:**
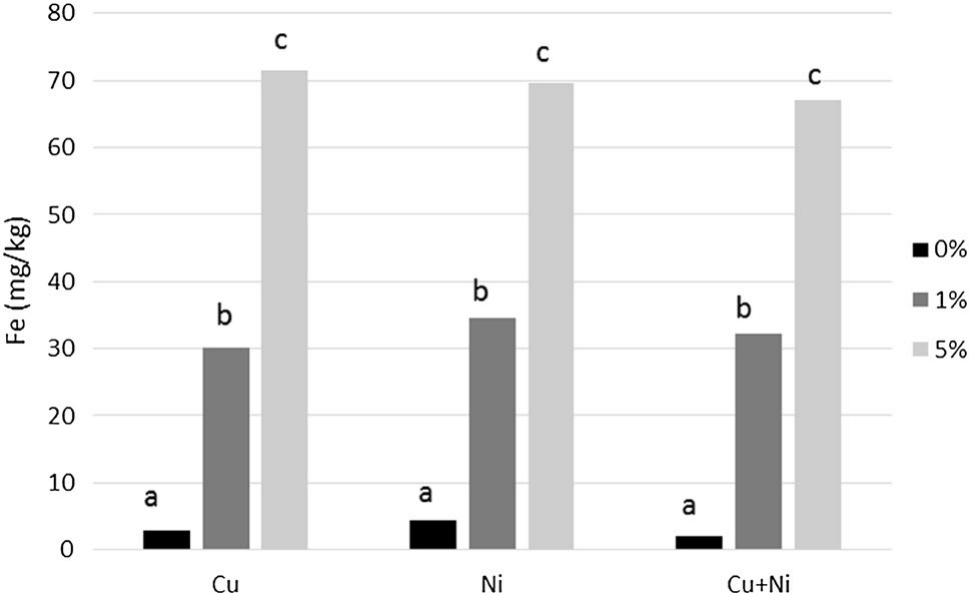
Mean concentration of Fe (mg/kg) in TCLP extract. For each polluted soil, bars followed by the same letter do not significantly differ (p < 0.05).

In parallel, the impact of nZVI addition on Fe availability was studied using the TCLP and Tessier extraction procedure. The Fe concentration in the TCLP extract increased (p < 0.001) with the dose of nZVI, from values lower than 5 mg/kg in control soils to 30 and 70 mg/kg at 1% and 5% of nZVI (Fig. 4). The Tessier extraction procedure showed that Fe was associated mainly with the residual fraction, followed by the oxide fraction (Table 2). The application of nZVI significantly increased the Fe associated with all fractions proportional to the dose of nZVI, especially in the EX + CB and OX fractions. The increase in Fe in the most available fractions (EX + CB) was dose-dependent, rising from values close to 10 mg/kg in control samples to 700 mg/kg in soils treated with 5% nZVI. The availability of this metal was predominately determined by soil pH. In the present study, the acidity of the soil favored Fe mobility, whereas other studies with neutral or alkaline soils reported limited mobility of this metal^32,33^. These results highlight the importance of soil properties on the effectiveness of nanoremediation strategies. The increase in Fe availability, as well as in potentially available Fe fractions, is of great interest in the context of enhancing soil fertility after remediation.

**Table 2:** Mean concentration of Fe (mg/kg) in EX + CB, OX, OM and RS soil fractions for each treatment.

Soil	Dose nZVI (%)	EX + CB	OX	OM	RS
Cu	0	9.67 ± 0.58 a A	812 ± 72.7 a A	45.7 ± 18.2 a A	6603 ± 635 a A
	1	115 ± 4.04 b A	2417 ± 240 b A	99.7 ± 45.1 a A	7488 ± 241 ab A
	5	698 ± 26.9 c A	7064 ± 564 c A	307 ± 70.0 b A	8374 ± 460 b A
Cu + Ni	0	11.3 ± 1.15 a A	751 ± 46.4 a A	35.7 ± 6.03 a A	7149 ± 479 a A
	1	230 ± 17.6 b B	2207 ± 219 b A	92.7 ± 47.9 a A	7183 ± 242 a B
	5	681 ± 51.8 c A	7195 ± 361 c A	383 ± 87.1 b A	8448 ± 155 b A
Ni	0	7.97 ± 0.21 a A	498 ± 5.86 a A	39.8 ± 3.22 a A	7055 ± 557 a A
	1	288 ± 10.4 b B	2054 ± 218 b A	117 ± 48.2 a A	8223 ± 367 b B
	5	695 ± 22.1 c A	6330 ± 118 c A	389 ± 76.4 b A	9171 ± 109 b B

Means followed by the same letter do not differ significantly (p < 0.05); lower letters compare among doses (0, 1 and 5%) of the same type of contamination; upper letters compare different types of contamination (Cu, Ni, and Cu + Ni) at the same dose.

### Impact of nZVI on soil properties

Table 3 lists the physico-chemical properties of the soil samples. The soil pH increased as a result of the high pH of the nZVI suspension (pH close to 11). This observation is in agreement with previous studies which also reported an increase in soil pH after the addition of nZVI^24,26,33,34^. However, this increase depends on soil buffering capacity. Thus, in alkaline soils, with high buffering capacity, the addition of nZVI moderately increased the pH^15,22,29,34^. The electrical conductivity significantly decreased in the soils polluted with Ni and Cu + Ni after treatment with nZVI and this effect was dose-dependent, probably due to the reduction of metal availability. No significant changes related to these parameters were observed in Cu-polluted soils. This finding is attributed to the lower effectiveness of nZVI to remove Cu, compared to Ni. Available phosphorus significantly decreased as the dose of nZVI increased. This effect may be due to the capacity of nZVI to immobilize phosphate and other anions^35,36^. Available sodium (Na) was significantly enhanced as the dose of nZVI increased. This effect is attributed to the presence of a stabilizing agent in the commercial nanoparticles, namely the sodium salt of polyacrylic acid. However, the mean Na concentrations were in the normal range for soils. Previous experiments with the same type of nanoparticles also detected an increase in available Na^21,26,33^. In this regard, under field conditions in a long-term experiment, Gil-Díaz et al.^21^ observed that available Na increased after nZVI application, but decreased over time to reach the original concentration. These results show that the application of nZVI under our experimental conditions did not adversely affect soil properties, although they should be taken into account before designing a nanoremediation treatment.

**Table 3:** Physico-chemical characteristics of polluted soils treated with nZVI.

Parameter	Cu			Ni			Cu + Ni		
	Control	1% nZVI	5% nZVI	Control	1% nZVI	5% nZVI	Control	1% nZVI	5% nZVI
pH	4.63 ± 0.02 a	5.33 ± 0.16 b	5.98 ± 0.05 c	5.31 ± 0.02 a	5.26 ± 0.03 a	5.66 ± 0.01 b	4.58 ± 0.01 a	4.77 ± 0.01 b	5.24 ± 0.06 c
EC (dS/m)	1.36 ± 0.10 a	1.27 ± 0.12 a	1.21 ± 0.22 a	2.15 ± 0.07 c	1.97 ± 0.03 b	1.53 ± 0.07 a	2.97 ± 0.04 b	2.87 ± 0.11 b	2.03 ± 0.08 a
N (%)	0.06 ± 0.00 a	0.06 ± 0.01 a	0.06 ± 0.00 a	0.06 ± 0.00 a	0.06 ± 0.00 a	0.06 ± 0.00 a	0.06 ± 0.00 a	0.06 ± 0.00 a	0.06 ± 0.00 a
OM (%)	0.86 ± 0.08 a	0.91 ± 0.04 a	0.79 ± 0.02 a	0.85 ± 0.12 a	0.80 ± 0.06 a	0.75 ± 0.08 a	0.83 ± 0.01 a	0.71 ± 0.07 a	0.84 ± 0.12 a
P (mg/kg)	32.7 ± 1.2 c	21.0 ± 1.0 b	10.7 ± 0.6 a	20.3 ± 0.6 c	15.7 ± 1.2 b	12.3 ± 1.5 a	27.7 ± 1.5 b	15.3 ± 1.2 a	12.3 ± 1.2 a
Ca (mg/kg)	619 ± 140 a	829 ± 64.0 a	750 ± 45.0 a	835 ± 37 a	824 ± 40 a	769 ± 17 a	761 ± 179 a	676 ± 87 a	750 ± 45 a
Mg (mg/kg)	84.0 ± 8.7 a	90.3 ± 12.3 a	84.3 ± 9.0 a	85.7 ± 4.0 a	81.0 ± 2.0 b a	71.7 ± 6.1 a	87.7 ± 3.5 a	94.7 ± 3.2 a	78.7 ± 15.3 a
Na (mg/kg)	22.0 ± 2.6 a	49.3 ± 2.5 b	107 ± 14 c	21.7 ± 2.3 a	43.3 ± 1.2 b	86.7 ± 6.0 c	28.7 ± 5.5 a	48.3 ± 7.8 b	94.3 ± 8.3 c
K (mg/kg)	171 ± 9 a	185 ± 5 a	178 ± 8 a	180 ± 1 a	184 ± 8 a	164 ± 4 a	184 ± 5 a	185 ± 3 a	169 ± 8 a

EC, electrical conductivity; OM, organic matter. Means followed by the same letter do not significantly differ (p < 0.05).

### Impact of nZVI on soil phytotoxicity

**Figure 5:**
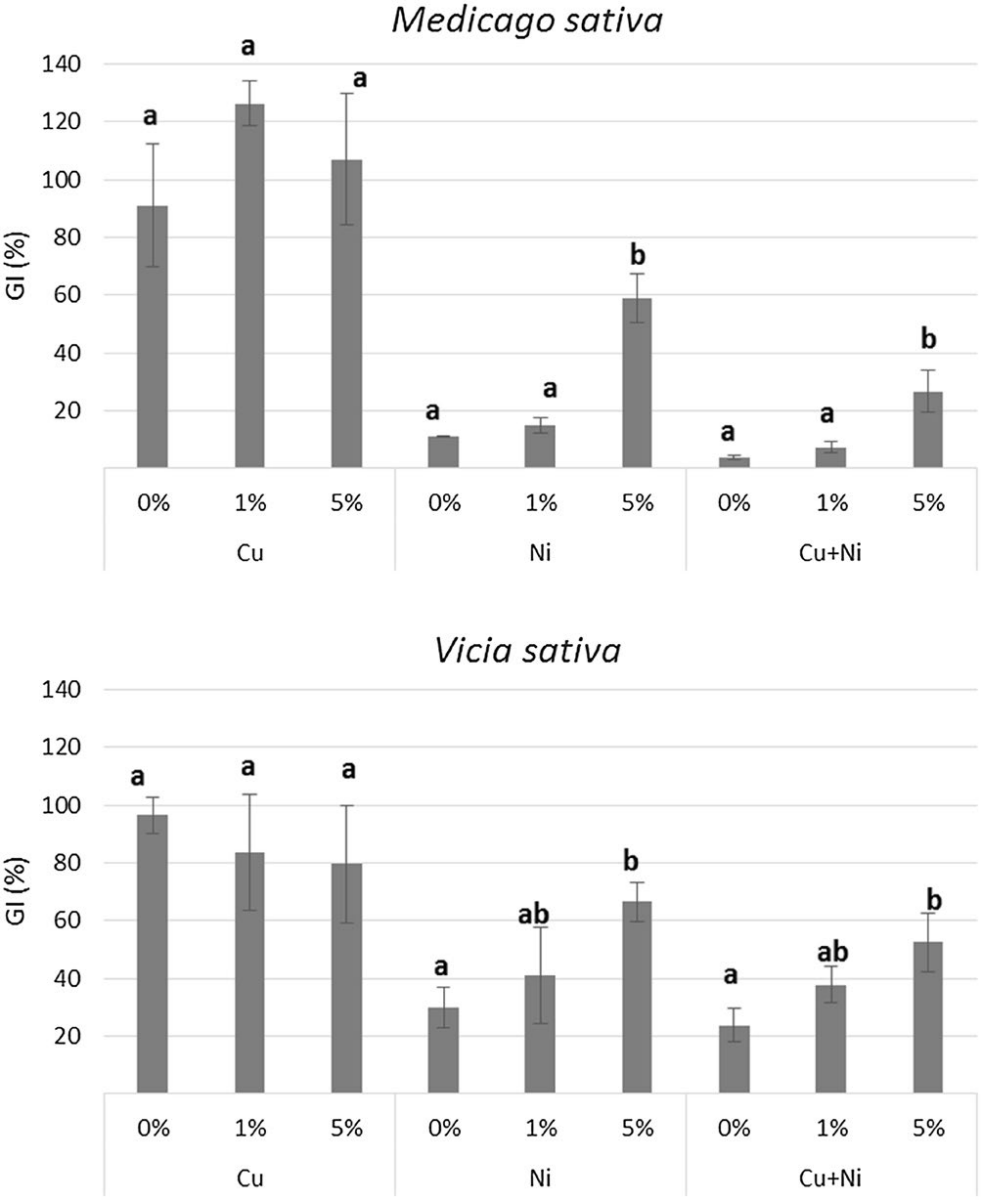
Mean germination index (%) of *Vicia sativa* and *Medicago sativa* for the soils at the different conditions. For each polluted soil, bars followed by the same letter do not significantly differ (p < 0.05).

Figure 5 shows the results of the phytotoxicity assay for *Vicia sativa* and *Medicago sativa*. According to Zucconi et al.^28^, GI values below 50% indicate high phytotoxicity, between 50 and 80% moderate phytotoxicity, and above 80% no phytotoxicity. Any phytotoxicity was shown in soils polluted with Cu regardless of the nanoparticle treatment. Thus, the application of nZVI did not induce phytotoxicity. In contrast, soils polluted with Ni showed high toxicity for both species, and the application of nZVI, especially at the highest dose, significantly reduced soil phytotoxicity. This result can be explained by the reduction of Ni availability achieved after the nanoremediation treatment. *Medicago sativa* was more sensitive to Ni contamination than *Vicia sativa*. Soils contaminated with Cu + Ni showed greater phytotoxicity compared to those contaminated with Ni alone. This is probably due to the lower capacity of nZVI to remove Ni in presence of Cu, thus implying higher Ni availability in soils. Previous experiments also detected a decrease in soil phytotoxicity after the reduction of As, Hg and Pb bioavailability by nZVI treatment^14,22,26^.

## Conclusions

The effectiveness of commercial nZVI for Cu and Ni immobilization in water and soil samples was dependent on the dose of nZVI used and the presence of both metals. Better immobilization results were achieved in water samples than in soil samples. The 5% dose of nZVI completely removed the two metals from water, even when the samples were co-contaminated. At a lower dose of nZVI (1%), the effectiveness decreased, achieving 64–72% of immobilized Cu and Ni in the case of single contamination. However, in Cu + Ni water samples, the removal of Cu was not affected by the presence of Ni, whereas the effectiveness of nZVI for Ni immobilization strongly decreased (23–31% immobilized) in co-contaminated samples and was not stable after 72 h. In soil samples, immobilization with 5% nZVI was more effective for Ni than for Cu, with a 54% and 21% reduction of leachability, respectively, in single contaminated samples. In soil samples contaminated with Cu + Ni, the nanoremediation treatment led to a significant decrease in Ni immobilization, as previously observed in water samples. With respect to the impact of nZVI on soil, a dose-dependent increase of available Fe was observed—an effect that is highly relevant in the context of soil rehabilitation. Regarding phytotoxicity, the germination assays with *Medicago sativa* and *Vicia sativa* seeds revealed that nZVI treatment did not induce phytotoxicity under the experimental conditions tested, and the phytotoxicity induced by Ni significantly decreased after applying nZVI. Accordingly, the use of nZVI emerges as an interesting option for Cu and/or Ni immobilization in water samples. In acidic soil samples, the effectiveness of nZVI to remove Cu was moderate, while for Ni it was strongly dependent on the presence of Cu. Thus, it would be inappropriate to extrapolate the results in water samples to soil samples. Further experiments, carried out using combined technologies over a longer period of time, would be necessary, in order to evaluate the stability of the process and to enhance the immobilization of Cu and Ni.
